# The respiratory microbiota alpha-diversity in chronic lung diseases: first systematic review and meta-analysis

**DOI:** 10.1186/s12931-022-02132-4

**Published:** 2022-08-23

**Authors:** Marta Avalos-Fernandez, Thibaud Alin, Clémence Métayer, Rodolphe Thiébaut, Raphaël Enaud, Laurence Delhaes

**Affiliations:** 1 University of Bordeaux, Bordeaux Population Health Research Center, UMR U1219, INSERM, F-33000 Bordeaux, France; 2 SISTM team Inria BSO, F-33405 Talence, France; 3 Pole of Public Health, University Hospital of Bordeaux, F-33000 Bordeaux, France; 4 Cystic fibrosis centre (CRCM), Paediatrics Department, University Hospital of Bordeaux, F-33000 Bordeaux, France; 5 Parasitology-Mycology Department, University Hospital of Bordeaux, F-33000 Bordeaux, France; 6 University of Bordeaux, Bordeaux Cardio-Thoracic Research Center, U1045, INSERM, F-33000 Bordeaux, France

**Keywords:** Human lung microbiome, Human lung bacteriome, Alpha-diversity, Chronic respiratory diseases, Asthma, Chronic obstructive respiratory disease, Cystic fibrosis, Non-cystic fibrosis bronchiectasis, Meta-analysis, Random-effects models, Factor Analysis of Mixed Data

## Abstract

**Background:**

While there seems to be a consensus that a decrease in gut microbiome diversity is related to a decline in health status, the associations between respiratory microbiome diversity and chronic lung disease remain a matter of debate. We provide a systematic review and meta-analysis of studies examining lung microbiota alpha-diversity in patients with asthma, chronic obstructive pulmonary disease (COPD), cystic fibrosis (CF) or bronchiectasis (NCFB), in which a control group based on disease status or healthy subjects is provided for comparison.

**Results:**

We reviewed 351 articles on title and abstract, of which 27 met our inclusion criteria for systematic review. Data from 24 of these studies were used in the meta-analysis. We observed a trend that CF patients have a less diverse respiratory microbiota than healthy individuals. However, substantial heterogeneity was present and detailed using random-effects models, which limits the comparison between studies.

**Conclusions:**

Knowledge on respiratory microbiota is under construction, and for the moment, it seems that alpha-diversity measurements are not enough documented to fully understand the link between microbiota and health, excepted in CF context which represents the most studied chronic respiratory disease with consistent published data to link alpha-diversity and lung function. Whether differences in respiratory microbiota profiles have an impact on chronic respiratory disease symptoms and/or evolution deserves further exploration.

**Supplementary Information:**

The online version contains supplementary material available at 10.1186/s12931-022-02132-4.

## Background

In the last decades, thanks to advancements in Next Generation Sequencing (NGS) technologies and bioinformatics, we have assisted to an explosion of studies on microbial communities in human bodies (i.e. human microbiome). The gut microbiome has been the most studied body site of the human microbiome. In particular, imbalance in its microbial composition (i.e. dysbiosis) is now considered an indicator of deteriorating health and has been associated with a variety of chronic health conditions such as obesity, type 2 diabetes, non-alcoholic liver disease, Crohn disease, and cardio-metabolic diseases [1–3].

One common indicator of dysbiosis is a modified (mainly a lower) overall microbial alpha-diversity, which denotes the relative abundance of microbial species in space and time within a given community (in practice, in the biological sample). In contrast, beta-diversity and gamma-diversity measure, respectively, the changes in species diversity between different communities and the overall diversity for the different communities within a region or over time. When quantifying alpha-diversity, richness and evenness are the main dimensions [4]. The former refers to the number of different species present in a given community. The later compares the uniformity of the population size of each of the species. Chao1, Shannon and Simpson’s diversity indexes are popular mathematical measures of species alpha-diversity in a community. Chao1 focuses on species richness. Shannon and Simpson’s indexes measure both species richness and evenness. Whilst Simpson’s strengthens evenness, Shannon strengthens richness. They are usually used to describe dysbiosis.

While there appears to be a consensus that a decreased alpha-diversity of the gut microbiome is linked to a declined health status [1–4], there is no clear evidence how this generalizes to the other microbiomes of the different human body sites. Especially, relationships between respiratory microbiota diversity and chronic lung diseases have been recently explored although microbial colonization of the airways tract has long characterized chronic lung diseases such as asthma, chronic obstructive pulmonary disease (COPD), cystic fibrosis (CF), or non-cystic fibrosis bronchiectasis (NCFB). In fact, the healthy lungs were long believed to be sterile (i.e. null diversity) after the forth bronchial division, which has clearly limited the interest of studying the lung microbial diversity until recently. In addition, compared to the gut microbiota, the lung microbiota is characterized by invasive access and uneasy collecting of samples (excepted collecting sputum samples), and low bacterial density in the samples connected to the upper respiratory samples [5, 6].

Chronic respiratory diseases, especially asthma and COPD, are a major cause of death and disability worldwide [7]. Throughout their respiratory illness course, patients experience stable phases, during which the illness course and symptoms are under control, punctuated by exacerbation phases, during which the illness suddenly becomes uncontrolled and symptoms increase resulting in an intensification of the provision of medical services to patients. By relying on NGS technologies, several studies were able to show that respiratory microbiota drastically changes during the occurrence of pulmonary pathologies [6, 8]. Identifying a range of values in alpha-diversity indexes when comparing exacerbated and stable patients with chronic respiratory diseases and healthy subjects may lead physicians to identify new biomarkers in chronic respiratory diseases.

In the present work, we propose a systematic review of studies investigating the lung microbiota alpha-diversity in patients with chronic respiratory diseases in which a control group based on disease status or healthy subjects is provided for comparison. We focused on the most common measures of alpha-diversity (Chao1, Shannon, and Simpson indexes) of the most frequently measured microbiome component (bacteriome), and the most common chronic diseases (asthma, COPD, CF, NCFB, and pulmonary hypertension [9]). Subsequently, we conducted a meta-analysis based on random-effects models to characterize (whenever possible) the difference in alpha-diversity indexes when comparing cases to controls. We explored sources of heterogeneity and assessed quality and bias risk. Finally, we discussed potential clinical relevance of lung microbiota alpha-diversity metrics. To the best of our knowledge, this is the first meta-analysis focused on alpha-diversity of lung microbiota associated with the most common chronic diseases.

## Methods

### Protocol and registration

We conducted a systematic review according to the recommended ”Preferred Reporting Items for Systematic Reviews and Meta-analyses” (PRISMA) guidelines that incorporate network meta-analysis [10, 11] and “Meta-analysis of Observational Studies in Epidemiology” (MOOSE) consensus statement [12]. This systematic review has been registered in PROSPERO International Prospective Register of Systematic Review (CRD42020140990).

### Data source and search strategies

We limited the search to the five most common chronic respiratory diseases known to be associated to microbial colonization/infections [6, 8, 9]: asthma, COPD, CF, NCFB, and pulmonary hypertension. The search was conducted using PubMed, Medline and Scopus databases, and was last updated on September 2021 using the equations summarized in Table 1.

**Table 1:** Equations used to search for articles within databases

Databases	Equations used
Pubmed/Medline	(microbiome*[Title/Abstract] OR microbiota[Title/Abstract] OR mycobiome*[Title/Abstract] OR mycobiota[Title/Abstract] OR virome[Title/Abstract] OR flore*[Title/Abstract] OR flora[Title/Abstract] OR microflor*[Title/Abstract] OR microbiota[MeSH Terms]) AND (diversity[Title/Abstract]) AND (asthma*[Title/Abstract] OR Asthma[MeSH Terms] OR COPD[Title/Abstract] OR ”chronic obstructive pulmonary disease”[Title/Abstract] OR ”Hypertension, Pulmonary”[MeSH Terms] OR ”cystic fibrosis”[Title/Abstract] OR ”Cystic Fibrosis”[MeSH Terms] OR bronchiecta*[Title/Abstract] OR ”pulmonary arterial hypertension”[Title/Abstract] OR ”Pulmonary Disease, Chronic Obstructive”[MeSH] OR lung disease*[Title/Abstract] OR bronchopulmonary disease*[Title/Abstract] OR pulmonary disease*[Title/Abstract] OR airways disease*[Title/Abstract])
Scopus	((TITLE-ABS(microbiome*) OR TITLE-ABS(microbiota) OR TITLE-ABS(mycobiome*) OR TITLE(mycobiota) OR TITLE-ABS(virome) OR TITLE-ABS(flore*) OR TITLE-ABS(flora) OR TITLE-ABS(microflor*)) AND (TITLE-ABS(diversity)) AND (TITLE-ABS(asthma*) OR TITLE-ABS(COPD) OR TITLE-ABS(”chronic obstructive pulmonary disease”) OR TITLE-ABS(”cystic fibrosis”) OR TITLE-ABS(bronchiecta*) OR TITLE-ABS(”pulmonary arterial hypertension”) OR TITLE-ABS(”lung disease*”) OR TITLE-ABS(”bronchopulmonary disease*”) OR TITLE-ABS(”pulmonary disease*”) OR TITLE-ABS(”airways disease*”)))

Finally, to ensure the comprehensiveness of the literature search, a second search strategy was then performed. The “backward snowballing” method was applied to identify relevant articles not identified by the search equations from the reference lists of the included studies.

### Study selection and assessment of study quality

Articles had to meet the following criteria to be included in the review:

- Dealing with at least one of the chronic respiratory diseases studied;
- Exhibiting alpha-diversity indexes in the article or in the additional files that can be properly collected (from a table, boxplot or bar chart);
- Being an article where there is at least one control group (healthy or patients with a stable disease) and at least one case group (stable patients or exacerbated patients or all diseased patients);
- Including human adults (people $\ge$18 years-old from all origin, sex and age were included in this meta-analysis); since the diversity of children microbiome, under development, is more unstable and not comparable to those of adults, studies focused on children population were excluded.

The literature was collectively selected (TA, MAF, CM, RE, and LD) based on the above eligibility criteria. For comparability purposes, we focused on the most common measures of alpha-diversity: Chao1, Shannon, and Simpson indexes of the most frequently measured microbiome component: bacteriome. First, articles were selected on title and abstract, and then on full reading. All the authors agreed the final selection.

### Data collection process

After selection and transferring databases search results to the Zotero software, data were extracted from each article using a self-designed data extraction form. We collected the following data from eligible studies: i) study characteristics (title, first author’s name, year of publication, country and continent where the study was conducted, journal in which the study was published), ii) population characteristics (chronic respiratory disease involved at different degrees of severity, measured outcome, type and objectives of the study, sample sizes and patient age), iii) NGS method characteristics (type of respiratory samples, DNA extraction method, sequencing strategy used: metataxonomy or whole genome shotgun sequencing, alpha-diversity metrics, taxonomic levels selected, and normalization or rarefaction method used), iv) microbiota characteristics (main results, covariates, comorbidities and confusion factors associated, conclusive remarks especially regarding alpha-diversity indexes).

### Statistical analyses

#### Estimating the differences between cases and controls

When the mean and the standard deviation (SD) of the alpha-diversity indexes for cases and controls were not available, we instead collected the median and first and third quartiles and/or minimum and maximum values and estimated the mean and SD using the Box-Cox method, which does not rely on the assumption of normality [13]. We used the website graphic user interface [13]. When a study reported results separately by subgroups (other than cases-controls and respiratory diseases), we combined them into a single group [14]. The standardized mean and confidence interval ($95\%$ IC) for the difference in alpha-diversity index values between each group were calculated with the R package metafor [15], assuming heteroscedastic population variances.

#### Random-effects meta-analysis models

To summarize the information from the different studies, we assumed that differences in alpha-diversity indexes between cases and controls vary from one study to other. Random effects models (through the R package metafor) allow us to estimate pooled mean differences and $95\%$ IC and present them in a forest plot. We separately analyzed Chao1, Shannon and Simpson indexes, for each of the chronic respiratory diseases and for each of the type of samples. The random effects models were first applied to studies on the same type of samples and disease, secondly applied to studies on the same disease and finally to all the studies. Each model consisted of a fixed intercept, a fixed effect of type of case/control comparison and a random intercept term to describe variation among studies. In the present meta-analysis, the possible types of case/control comparisons were: patients with a stable chronic pulmonary disease vs. healthy people, patients with an exacerbation of the chronic pulmonary disease vs. healthy people, diseased patients vs. healthy group, and patients with an exacerbation of the chronic pulmonary disease vs. patients with a stable chronic pulmonary disease. The ANOVA test was used to evaluate the comparison group effect. Heterogeneity analysis was assessed through the Cochran’s Q test and the Higgins’ $I^2$ statistic analysis [16, 17].

### Heterogeneity sources’ exploration

Differences in study populations, samples, microbiome sampling techniques and protocols, and other study characteristics are potential sources of heterogeneity. We conducted a Factor Analysis of Mixed Data (FAMD) using the FactoMineR R package [18] to investigate whether the discrepancies between the results of the studies in term of alpha-diversity metrics were due to heterogenous experimental conditions or to inherent variability in the lung microbiota.

FAMD is a multivariate technique that analyzes data in which observations are described by several inter-correlated quantitative and qualitative variables. Thus, FAMD combines Principal Component Analysis for continuous variables and Multiple Correspondence Analysis for categorical variables. The goal is to extract the important information from the multivariate characteristic and represent it graphically. The sample size of cases and controls constitute the two continuous variables, and the type of samples (bronchoalveolar lavage (BAL), sputum, induced sputum, lower airways (LA), upper airways (UA)), the samples origin continent (Asia, America, or Europe), the NGS sequencing method (Pyrosequencing such as 454 FLX (Roche$^{\copyright }$) or bridge amplification such as MiSeq or HiSeq (Illumina$^{\copyright }$) or long read sequencing such as PacBio (Pacific Biosciences$^{\copyright }$), the use of rarefaction analysis (yes or no/not stated clearly), the taxonomic level used (OTU or genus level), and clustering method (OTU or ASV) constitute the categorical variables. The output of the analysis was a biplot projection in which similar studies (with respect to the listed variables) were close. Then, we visually evaluated whether the differences or similarities in alpha-diversity indexes between studies could be explained by distance or closeness in the FAMD biplot projection.

FAMD is an explanatory analysis where no statistical inference can be made. The ANOVA test was applied to assess the effect of study characteristics on alpha-diversity. As before, we analyzed Shannon, Chao1, and Simpson indexes separately and assumed random-effects meta-analysis models.

### Quality and risk of bias assessment

In parallel, quality assessment was performed independently by two authors (LD and RE). Studies were rated 1/3 (poor quality), 2/3 (average quality) or 3/3 (good quality) based on clinical characterization of the chronic lung diseases, case and control sample sizes, case and control comparability, microbiome sampling techniques, NGS procedures, and the sequencing methods and taxonomic levels. The final score was obtained as the sum of the two authors’ rate. The spacial distribution of the studies on the FAMD biplot was interpreted in the light of the quality score. Then, to assess risk of bias, we restricted the evaluation of differences in alpha-diversity indexes in the biplot to high quality studies. A similar bias assessment approach in meta-analysis has been previously proposed [19]. The ANOVA test was also applied to assess the effect of quality score on alpha-diversity assuming random effects models.

## Results

### Study selection

**Fig. 1:**
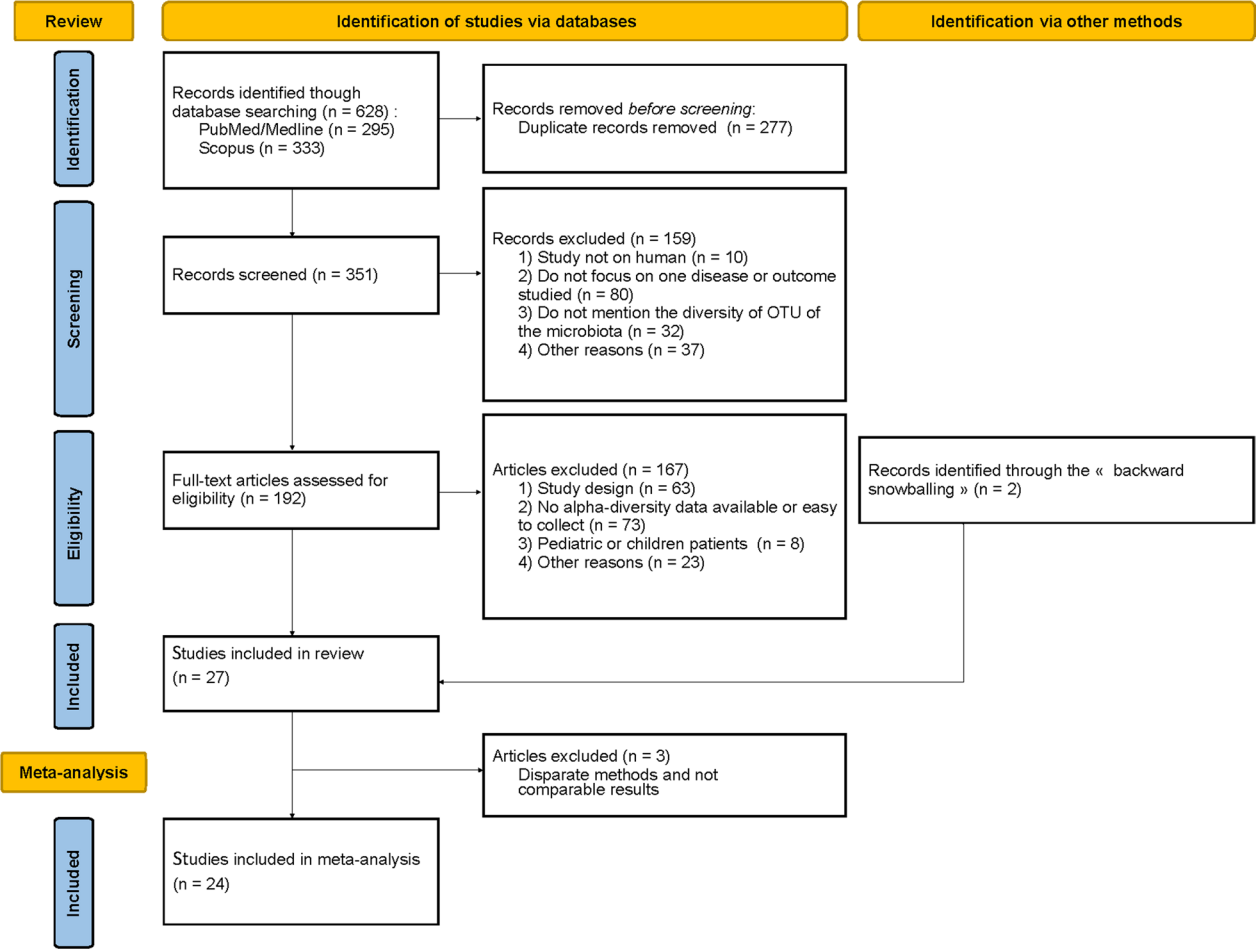
PRISMA flow diagram summarizing our search results and study selection for the systematic review and meta-analysis

Our search identified 628 articles in all, and after removing duplicate records ($n = 277$), we screened 351 articles on titles and abstracts. Among them, 160 articles were excluded at this stage mainly because they were not focused on disease or outcome studied here ($n = 80$), not reported alpha-diversity of microbiota ($n = 32$), or not on human microbiota ($n = 10$) (Fig. 1). Of the 192 full text articles reviewed for eligibility, 25 studies met our inclusion criteria for the systematic review. In addition, two studies were included using the backward snowballing method, as these point two studies did not included our inclusion criteria in the title or abstract; the metataxonomy analysis or the alpha-diversity indexes being found in the full text or the additional files [20, 21]. In the end 27 studies were included in the literature review. The reasons for exclusion at every step are summarized in Fig. 1. Finally, data from 24 studies were used in the final meta-analysis, as three studies were excluded: one study was the only one measuring alpha-diversity with the Fisher’s index [22], one study analyzed the phages associated with the bacteria [23], and the other study used a different, non-comparable molecular method since the authors analyzed the metagenomics profiles by using terminal restriction fragment length polymorphism (T-RFLP) which is not a NGS approach [24].

### Studies characteristics

Characteristics of studies included in the meta-analysis are presented in Table 2. Six studies deal with CF, 8 with COPD, and 10 with asthma, as primary disease (several studies targeting more than one chronic pulmonary disease, Table 2). Only one selected study focused on NCFB, while none on pulmonary hyper-tension. All of the studies dedicated to the respiratory tract microbiota, mainly based on sputum samples analysis (spontaneously expectorated sputum $n = 8$, and induced sputum $n = 5$) but also 6 studies focused on BAL, 2 on upper airway and 3 on lower airway samples analysis.

On the molecular side, the majority of the studies used metataxonomy approaches. However, these metataxonomy methods exhibited numerous heterogeneities. First, NGS analyses were conducted on 454 Roche$^{\copyright }$ pyrosequencing platform ($n = 12$), or Illumina$^{\copyright }$ System (mainly MiSeq platform, $n = 8$), PGM Ion torrent$^{\copyright }$ ($n = 2$), 454 Roche$^{\copyright }$ pyrosequencing plus Illumina$^{\copyright }$ ($n = 1$), or on Illumina$^{\copyright }$ plus PacBio Pacific Biosciences$^{\copyright }$ ($n = 1$) platforms. Two studies [20, 25] used shotgun whole genome sequencing, while the others utilized 16S sequencing. Two studies [21, 25] used the ASV approach for clustering the data. We also noted heterogeneity regarding the amplified region of the 16S rDNA, with the most widely used being the variable regions: V4 ($n = 5$ studies), V1-V3 ($n = 4$), V3-V5 ($n = 2$), V3-V4 ($n = 2$), and V1-V2 ($n = 2$). In addition, normalization procedure (especially the construction of the so-called rarefaction curves used to study diversity) was clearly mentioned and used in only 14 studies. In the remaining studies, construction of rarefaction curves was not mentioned.

On the clinical side, clinical status of patients, population sizes, and alpha-diversity indexes are detailed in Table 3. Shannon index was the most widely reported alpha-diversity index, reported across all the studies except [26], followed by Chao1 richness and Simpson indexes (reported in about one third of the studies). Whatever the NGS method and the index used, we noticed that the mean values were highly variable from one study to another (Table 3). Study designs also vary from study to study. Most studies use a case-control design, some studies use a cross-sectional design, and one study [27] uses a case-crossover design (i.e., cases and controls are the same subjects measured at two different time points).

**Table 2:** Characteristics of the studies selected for the meta-analysis (the samples origin continent: Asia, America, or Europe; type of samples: bronchoalveolar lavage (BAL), sputum, sputum (induced), lower airways (LA), upper airways (UA); the NGS sequencing method: 454 pyrosequencing for Pyrosequencing such as 454 FLX (Roche$^{{{\copyright }}}$), Illumina for bridge amplification such as MiSeq or HiSeq (Illumina$^{{{\copyright }}}$), 454 pyrosequencing/Illumina for combined methods, PacBio/Illumina for long read sequencing such as PacBio (Pacific Biosciences$^{{{\copyright }}}$) combined with Illumina, Other for the other combinations; the use of rarefaction analysis: YES or NO/NC (no or not stated clearly); the taxonomic level used: OTU or Genus (for OTU clustered at genus level); ASVs- vs

Study	Disease	Continent	Type of sample	NGS sequencing	Rarefaction	Level	ASV method	Design	$n_{0}$	$n_{1}$	Score
Goleva et al. [28]	Asthma	America	BAL	454 pyrosequencing	YES	Genus	NO ASV	Case-control	12	39	6
Denner et al. [29]	Asthma	America	BAL	Illumina	YES	Genus	NO ASV	Case-control	19	39	6
Sverrild et al. [30]	Asthma	Europe	BAL	Illumina	NO/NC	OTU	NO ASV	Case-control	10	23	5
Liu et al. [31]	Asthma	Asia	Sputum	Other	YES	OTU	NO ASV	Case-control	29	116	6
Li et al. [32]	Asthma	Asia	Sputum (induced)	454 pyrosequencing	NO/NC	OTU	NO ASV	Case-control	15	24	6
									15	25	
									24	25	
Marri et al. [33]	Asthma	America	Sputum (induced)	454 pyrosequencing	YES	OTU	NO ASV	Case-control	10	10	4
Huang et al. [34]	Asthma	Asia	Sputum (induced)	Illumina	YES	OTU	NO ASV	Case-control	16	22	6
Munck et al. [35]	Asthma	Europe	Sputum (induced)	454 pyrosequencing	YES	OTU	NO ASV	Case-control	20	44	6
Park et al. [36]	Asthma	Asia	UA	454 pyrosequencing	YES	Genus	NO ASV	Case-control	12	18	5
Lee et al. [20]	Asthma	Asia	UA	454 pyrosequencing/	NO/NC	OTU	NO ASV	Case-control	20	59	6
				Illumina							
Erb-Downward et al. [37]	COPD	America	BAL	454 pyrosequencing	NO/NC	OTU	NO ASV	Case-control	10	4	5
Pragman et al. [38]	COPD	America	BAL	454 pyrosequencing	YES	OTU	NO ASV	Case-control	10	22	5
Einarsson et al. [39]	COPD	Europe	LA	Illumina	NO/NC	OTU	NO ASV	Case-control	19	18	6
Kim et al. [40]	COPD	Asia	LA	454 pyrosequencing	YES	OTU	NO ASV	Case-control	13	13	6
Feigelman et al. [25]	COPD	Europe	Sputum	Illumina	NO/NC	Genus	ASV	Case-control	4	4	5
Millares et al. [41]	COPD	Europe	Sputum	454 pyrosequencing	NO/NC	OTU	NO ASV	Cross-sectional	8	8	4
Wang et al. [21]	COPD	Asia	Sputum (induced)	PacBio/Illumina	YES	OTU	ASV	Case-control	27	98	6
Park et al. [36]	COPD	Asia	UA	454 pyrosequencing	YES	Genus	NO ASV	Case-control	12	17	5
Pletcher et al. [42]	CF	America	LA	Illumina	YES	OTU	NO ASV	Case-control	17	9	5
Soret et al. [43]	CF	Europe	Sputum	454 pyrosequencing	YES	OTU	NO ASV	Case-control	16	17	5
Narayanamurthy et al. [44]	CF	America	Sputum	Illumina	NO/NC	Genus	NO ASV	Case-control	8	16	5
Filkins et al. [26]	CF	America	Sputum	454 pyrosequencing	NO/NC	Genus	NO ASV	Cross-sectional	22	13	6
Coburn et al. [45]	CF	America	Sputum	Illumina	YES	OTU	NO ASV	Cross-sectional	100	27	6
Carmody et al. [27]	CF	America	Sputum	454 pyrosequencing	YES	OTU	NO ASV	Self-controlled	34	34	6
Byun et al. [46]	NCFB	Asia	BAL	Other	NO/NC	Genus	NO ASV	Cross-sectional	8	6	3

OTUs-based approaches: ASV or No ASV; the study design; control group sample size ($n_{0}$); case group sample size ($n_{1}$); and the quality score)

**Table 3:** Characteristics of the samples from the studies selected for the meta-analysis relative to the groups used as cases and controls (”healthy”, ”stable”, ”exacerbated” and, when exacerbated and stable patients are mixed in one group or when the disease status is not reported, ”diseased”)

Study	Mean ± SD alpha-diversity index (sample size)											
	Shannon				Chao1				Simpson			
	Healthy	Stable	Exacerbated	Diseased	Healthy	Stable	Exacerbated	Diseased	Healthy	Stable	Exacerbated	Diseased
Goleva et al. [28]	$2.8\pm 0.7 \,(12)$	$2.8\pm 0.5 \,(39)$			$21\pm 7 \,(12)$	$26\pm 8 \,(39)$						
Denner et al. [29]	$3.5\pm 0.3 \,(19)$			$3.5\pm 0.2 \,(39)$	$158\pm 32 \,(19)$			$143\pm 36 \,(39)$	$0.92\pm 0.02 \,(19)$			$0.91\pm 0.02 \,(39)$
Sverrild et al. [30]	**3.8** ± **0.3** (10)			**4.1** ± **0.2** (23)								
Liu et al. [31]	$5.6\pm 0.7 \,(29)$	$6.0\pm 1.1 \,(116)$										
Li et al. [32]	$2.8\pm 0.5 \,(15)$	$2.8\pm 0.4 \,(24)$	$2.8\pm 0.5 \,(25)$		$181\pm 37 \,(15)$	$243\pm 149 \,(24)$	$229\pm 162 \,(25)$		$0.15\pm 0.12 \,(15)$	$0.13\pm 0.08 \,(24)$	$0.12\pm 0.03 \,(25)$	
Marri et al. [33]	$2.5\pm 0.3 \,(10)$	$2.8\pm 0.3 \,(10)$										
Huang et al. [34]	**3.3** ± **0.6** (16)			**2.9** ± **0.4** (22)	**571** ± **464** (16)			**271** ± **50** (22)	**0.11** ± **0.06** (16)			**0.13** ± **0.06** (22)
Munck et al. [35]	**2.7** ± **0.3** (20)			**3.2** ± **0.3** (44)								
Park et al. [36]	**3.5** ± **0.7** (12)	**2.4** ± **1.0** (18)			**274** ± **147** (12)	**173** ± **101** (18)						
Lee et al. [20]	**1.7** ± **0.6** (20)	**2.1** ± **1.2** (59)			**284** ± **208** (20)	**351** ± **383** (59)						
Erb-Downward et al. [37]	**3.6** ± **1.1** (10)	**3.1** ± **1.6** (4)										
Pragman et al. [38]	**0.5** ± **0.4** (10)	**1.6** ± **0.9** (22)							**0.27** ± **0.24** (10)	**0.61** ± **0.28** (22)		
Einarsson et al. [39]	**2.6** ± **0.5** (19)	**1.9** ± **0.6** (18)										
Kim et al. [40]	**2.1** ± **0.6** (13)			**1.8** ± **0.8** (13)					**0.3** ± **0.2** (13)			**0.4** ± **0.2** (13)
Feigelman et al. [25]	**3.1** ± **0.1** (4)			**1.5** ± **0.8** (4)								
Millares et al. [41]		$2.9\pm 1.0 \,(8)$	$3.3\pm 1.1 \,(8)$			$138\pm 54 \,(8)$	$126\pm 31 \,(8)$					
Wang et al. [21]	$6.2\pm 1.4 \,(27)$	$5.5\pm 2.3 \,(98)$										
Park et al. [36]	**3.5** ± **0.7** (12)	**2.9** ± **1.0** (17)			**274** ± **147** (12)	**203** ± **127** (17)						
Pletcher et al. [42]	$4.2\pm 0.9 \,(17)$			$2.2\pm 1.1 \,(9)$								
Soret et al. [43]	$0.8\pm 0.6 \,(16)$		$1.4\pm 3.4 \,(17)$		$30\pm 15 \,(16)$		$26\pm 9 \,(17)$		$0.30\pm 0.16 \,(16)$		$0.70\pm 2.21 \,(17)$	
Narayanamurthy et al. [44]	$2.6\pm 0.1 \,(8)$			$1.7\pm 0.8 \,(16)$	$134\pm 11 \,(8)$			$103\pm 18 \,(16)$				
Filkins et al. [26]										$0.64\pm 0.15 \,(22)$	$0.40\pm 0.48 \,(13)$	
Coburn et al. [45]		$4.1\pm 1.2 \,(100)$	$3.4\pm 1.6 \,(27)$									
Carmody et al. [27]		$1.3\pm 0.6 \,(34)$	$1.4\pm 0.6 \,(34)$									
Byun et al. [46]		$4.6\pm 12.9 \,(8)$	$0.5\pm 0.5 \,(6)$							$3.60\pm 6.57 \,(8)$	$1.77\pm 1.30 \,(6)$	

Values in bold are as reported in the original papers, plain text values were estimated from the quantiles in the papers

### Differences in alpha-diversity indexes between healthy and diseased people were more marked in CF.

**Fig. 2:**
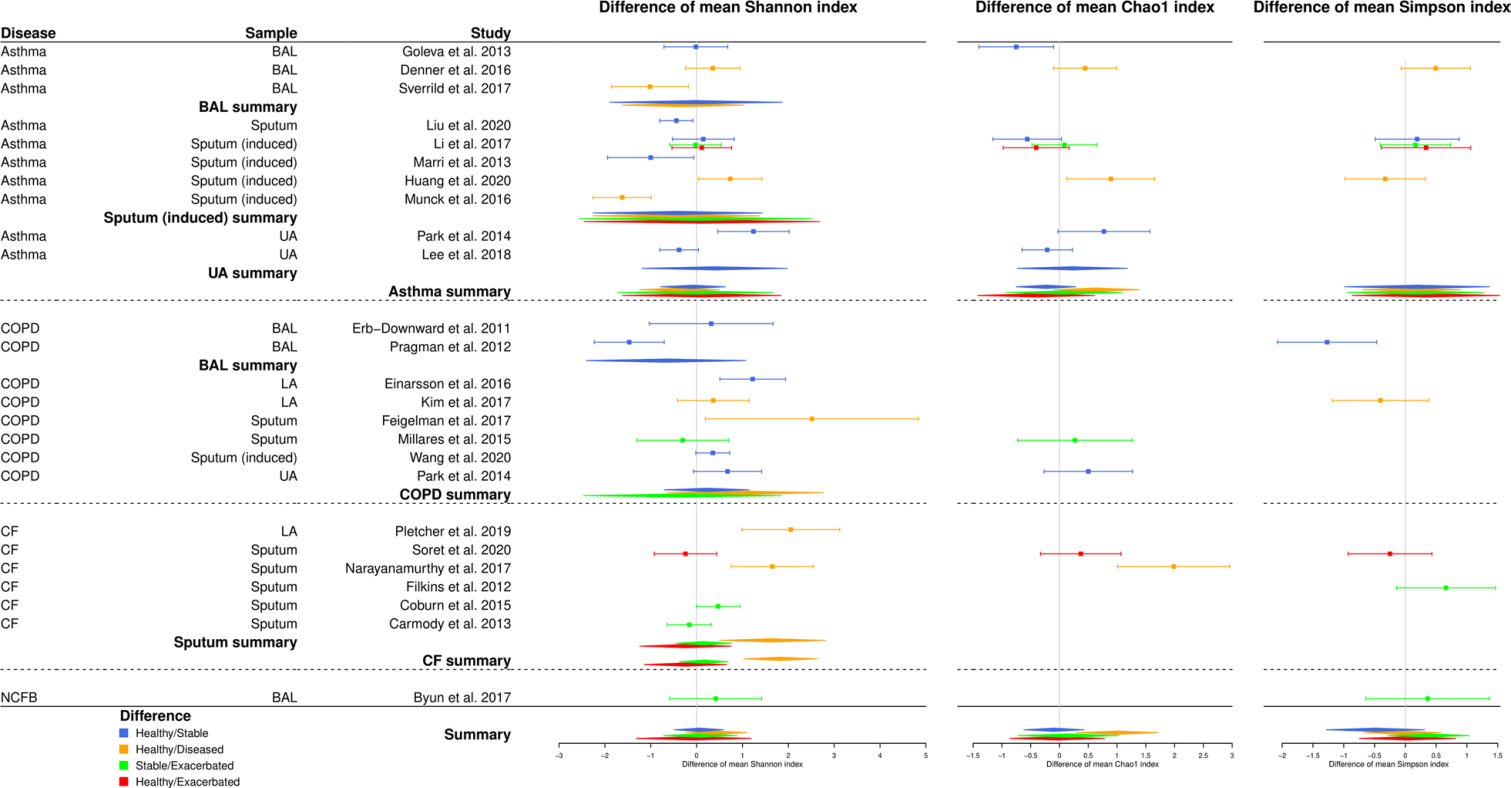
Forest plot summarizing results from the random-effects meta-analysis model. A summary by type of sample, by disease and for all the studies is estimated by assuming the random effects model when data are available for at least two comparable studies. Values to the right of the vertical axis (positive values) indicate that the diversity of the control group (the healthiest group in each comparison) is greater than that of the case group. Conversely, values to the left of the vertical axis (negative values) indicate that the diversity of the control group is lower than that of the case group. When a confidence interval crosses the vertical axis, the standardized difference between the mean value of control diversity and the mean value of case diversity is not significant for the given study

For each alpha-diversity index, each chronic respiratory disease, and each type of sample, we summarized the difference between cases and controls alpha-diversity values in a forest plot representation. Fig. 2 shows standardized differences between mean values for controls and mean values for cases and their confidence intervals.

Half of the studies provided no significant difference in alpha-diversity indexes between controls and cases. Some studies exhibited a significant increase of alpha-diversity in cases: for asthma via the Shannon and Chao1 indexes, when comparing healthy and stable patients [28, 31, 33] and healthy and diseased patients [30, 35]; for COPD via the Shannon’s and Simpson’s indexes when comparing healthy and stable or exacerbated patients [38].

On the other side, few studies provided a significant increase of alpha-diversity indexes in controls: for asthma via Shannon index when comparing healthy and stable patients [36], and via Shannon and Chao1 indexes when comparing healthy and diseased patients [34]; for COPD via the Shannon index, when comparing healthy and stable patients [39] and healthy and diseased patients [25]; for CF, when comparing healthy with diseased patients [42, 44] and stable with exacerbated patients [45] (via Shannon for all these three studies, besides Chao1 index for [44]).

Some studies presented opposite findings when using different alpha-diversity measures. For example, alpha-diversity in healthy people has a tendency to be higher than in stable or exacerbated asthmatic patients, using Shannon or Simpson indexes and lower when using Chao1 index [32]. In COPD, higher alpha-diversity among healthy people compared to diseased patients was observed using Shannon index and lower when using Simpson index [40]. On the other hand, lower Shannon index values and higher Chao1 index values were observed in stable compared with exacerbated patients [41].

In CF, the Shannon and Chao1 diversities of healthy individuals appeared to be consistently higher than those of CF patients (yet this result is based on only two studies [42, 44], only one for the Chao1 index [44]).

The effect of the comparison group (ANOVA tests, Table 4) was not significant except for CF with the Shannon index (considering only sputum samples, $p=0.04$, as well as considering all samples together, $p=0.0007$). The analysis of the Cochran’s Q test and the Higgins $I^2$ statistics confirmed that substantial heterogeneity was present, except for CF with the Shannon index and for asthma with the Simpson index (Table 4). We documented further this heterogeneity using quality and risks of bias assessment.

**Table 4:** Random-effects model statistics: p-values for ANOVA tests (used to assess the effect of comparison group), p-values for Cochran’s Q tests and Higgins’ $I^2$ statistics (both used to assess heterogeneity)

Alpha-diversity index	Disease	Sample type	ANOVA p-value	Cochran’s Q p-value	$I^2$ statistic
Shannon	Asthma	BAL	0.81	0.01	$85\%$
		Sputum (induced)	0.98	$< 0.01$	$92\%$
		UA	–	$<0.01$	$92\%$
		All	0.94	$< 0.01$	$87\%$
	COPD	BAL	–	0.02	$80\%$
		All	0.59	$< 0.01$	$87 \%$
	CF	Sputum	0.04	0.07	$70\%$
		All	$<0.01$	0.16	$48 \%$
	NCFB		–	–	–
	All		0.82	$< 0.01$	$86\%$
Chao1	Asthma	UA	–	0.03	$78\%$
		All	0.24	0.03	$65 \%$
	COPD		–	–	–
	CF		–	–	–
	NCFB		–	–	–
	All		0.09	$<0.01$	$68 \%$
Simpson	Asthma	All	0.99	0.06	$71\%$
	COPD		–	–	–
	CF		–	–	–
	NCFB		–	–	–
	All		0.45	0.02	$59 \%$

A line indicates that the test could not be performed (since only one comparison group, in the case of ANOVA, or insufficient number of studies, in the case of heterogeneity statistics)

### Quality and risk of bias assessment of the meta-analysis

As Shannon index was the most reported metric throughout the 24 selected studies (Fig. 2 and Table 3), we focused on this metric and explored the corresponding heterogenity using FAMD approach.

The percentage of variance explained by the first two factors is about $32\%$. Each study position depends on its population and sampling characteristics (Additional file 1: Fig. S4). In addition, the distance between studies depends on the closeness of their characteristics (Additional file 1: Figs. S5−S12). The quality assessment performed by two experts (Table 2) did not allow to characterize the spacial distribution of the studies in the biplot (Fig. 13, Supplementary material).

**Fig. 3:**
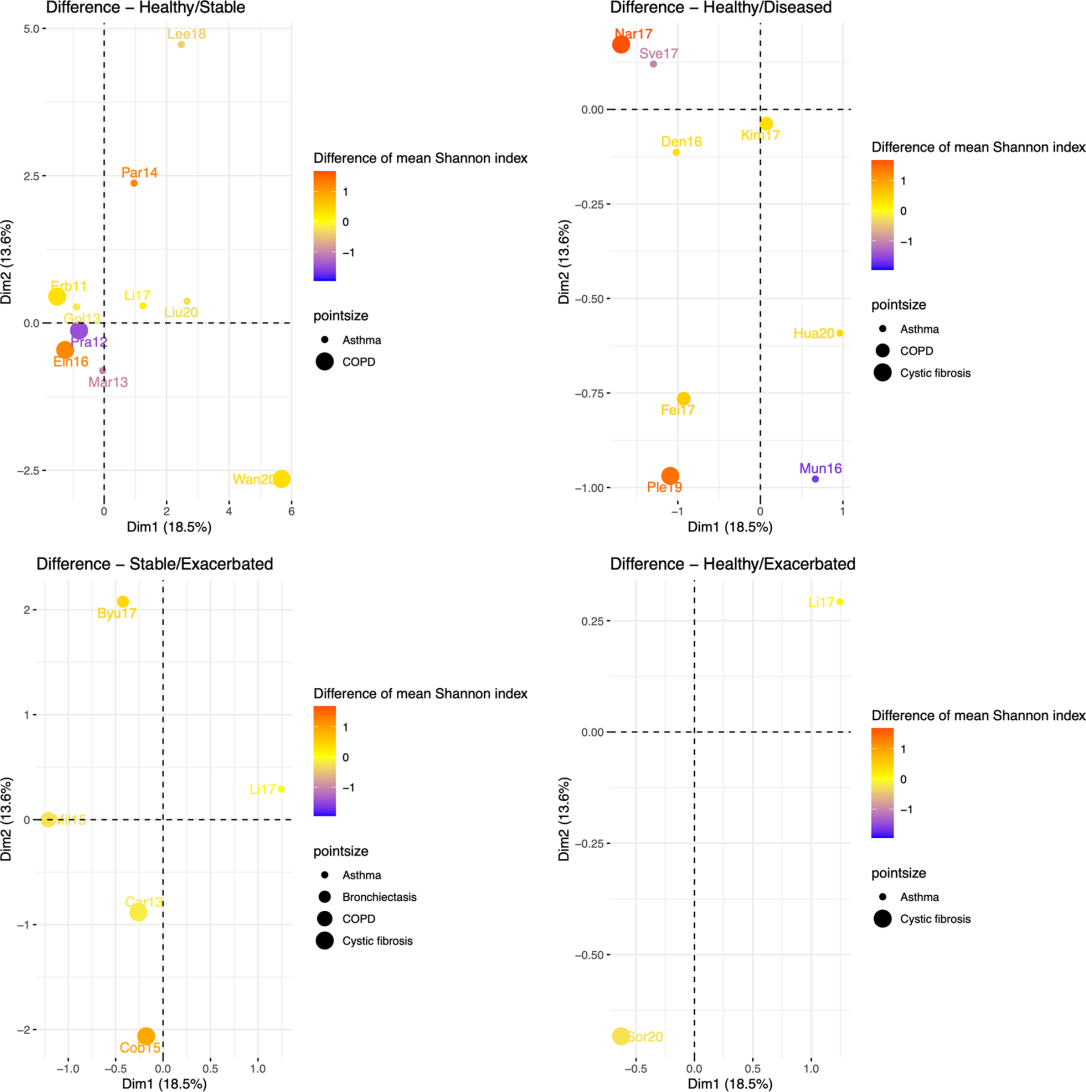
FAMD biplot Vs. mean Shannon diversity differences between cases and controls. Dot sizes of studies (Byu17 [46] Car13 [27], Cob15 [45], Den16 [29], Ein16 [39], Erb11 [37], Fei17 [25], Gol13 [28], Hua20 [34], Kim17 [4], Lee18 [20], Li17 [32], Liu20 [31], Mar13 [33], Mil15 [41], Mun16 [35], Nar17 [44], Par14 [36], Ple19 [42], Pra12 [38], Sor20 [43], Sve17 [30], Wan20 [21]) are different depending on the disease. Color degree represents the sign (positive or negative) and the amount of the difference between mean diversity of cases and mean diversity of controls

Then, we explored the biplot of FAMD as a function of the mean Shannon diversity differences between cases and controls (Fig. 3). Whatever the respiratory disease (asthma, COPD, CF, or NCFB), the distribution according to the color degrees (between 1 in red to -1 in blue) appeared to have a random distribution. For example, when comparing the difference in Shannon diversity between healthy individuals and stable COPD patients, Pragman et al. [38] and Einarsson et al. [39], close in the FAMD biplot (which indicates close study conditions) show opposite results (higher diversity in cases in the first study while lower in the second study) (Fig 3). Inversely, when comparing healthy individuals and diseased CF patients, Narayanamurthy et al. [44] and Pletcher et al. [42], distant in the FAMD biplot (which indicates quite different study conditions) show similar results (higher Shannon diversity in controls). The same trends were observed with Chao1 and Simpson indexes (results not shown). We come to the same conclusion when restricting to top quality studies. Finally, we assessed the effect of characteristics on the difference in Shannon values between cases and controls (Additional file 1: Table S5). The quality of studies (measured by the sum of expert assessments) did not appear to be related to the difference observed between cases and controls. Differences observed in asthma studies appeared to be associated to the samples origin continent (3 American, 2 European and 5 Asian studies), while the NGS sequencing method appeared to be associated in CF studies (half of the studies used Illumina and the other half, 454 pyrosequencing). Differences in Shannon diversity appeared to be linked to the taxonomic level when considering all the diseases altogether. The type of samples did not show any association. These results from ANOVA tests should be taken with caution because no adjustments are made for the type of comparison between cases and controls or other variables and, in addition, the number of studies is small.

## Discussion

In this systematic review and meta-analysis, we highlighted that when looking at the big picture (all diseases combined, asthma, COPD, CF and NCFB; for all alpha-diversity measures, Shannon, Chao1 and Simpson indexes), there is no clear trend in the respiratory microbiota diversity of people with some chronic respiratory diseases (healthy vs. stable/ill/exacerbated) compared with that of healthy people neither in individuals with chronic lung disease during a stable period compared with individuals with chronic lung disease during an acute exacerbation (Fig. 2). However, we found a slight trend toward greater respiratory microbiota Shannon diversity in stable patients compared with exacerbated patients and a more marked trend toward greater respiratory microbiota Chao1 diversity in healthy vs. diseased individuals. Yet, these results should be interpreted with caution given the limited number of studies available.

Looking closely at each chronic respiratory disease, we have shown that in CF there is a greater respiratory microbiota diversity in healthier individuals (healthy vs. ill) (Fig. 2) and a slight trend toward greater respiratory microbiota diversity in stable vs. exacerbated patients. Results from the analysis of alpha-diversity for healthy people vs. asthmatic or COPD people appeared to be more mitigated, in line with published data exhibiting divergent conclusive remarks [38, 41, 47]. Furthermore, the absence of significant difference in respiratory microbiota profiles between mild and moderate COPD patients and healthy people has been noticed recently [48]. In addition, the lung microbiota is known to display greater spatial variation between individuals than within individuals and chronic respiratory diseases, especially COPD and asthma, are recognized as highly heterogeneous diseases. As COPD is classically considered to be a bronchial inflammation in which neutrophils play a central role while asthma is more particularly associated with eosinophilic airway inflammation [49], it is difficult to compare these different populations and their lung microbiota, especially regarding alpha-diversity metrics [50, 51]. We compared alpha-diversity metrics by type of indexes for the different chronic respiratory diseases: asthma, COPD, CF and NCFB, but most of the studies included in this meta-analysis are cross-sectional studies. Even if a continuum has been proposed between several chronic respiratory diseases (especially from asthma to COPD [52]), these diseases remains clinical entities with an adapted therapeutic management and numerous heterogeneities between them [50, 51]. In addition, recent published data demonstrated that the microbiome composition and its alpha-diversity indexes at a unique single time-point could not classify CF patients in ”stable” and ”decliner”, for example [53]. To overcome these biases, longitudinal studies are warranted, as recently proposed [53].

While understanding the drivers of diversity remains a key point in ecology, there are different methods and parameters for describing diversity and documenting its effects on ecosystem health and function. Among them, alpha-diversity indexes, especially Shannon, Chao1 and Simpson’s indexes, are widely used to described the diversity at the local (the biological sample) scale. Whilst Simpson’s strengthens evenness, Shannon strengthens richness. Moreover, Shannon index is a type I index that is sensitive to important variations of the rarest species, but Simpson index belongs to type II indexes, sensitive to major variations of the most abundant species. Albeit Shannon index remains the most common alpha-diversity index, several other indexes can be used. For example, Fisher’s alpha index, which refers to Fisher’s logarithmic series model, represents the first attempt to describe mathematically the relationship between the number of species and the number of individuals in those species, and has been successfully used to demonstrate that microbiota diversity, dominance, and the identity of the dominant bacterial species are informative indicators of CF disease state in combination with measures of lung function in a multicentric study [22]. In addition, the concept of microbial translocation in the CF airways has been proposed and documented [42], which highlights the specificity of alpha-diversity measures to a given site and disease. The Berger-Parker dominance index, which measures the proportion of the microbiome dominated by the most abundant taxa, has also been applied in respiratory research [54]. This index focuses on dominant species rather than rare species and can provide important additional information when the study focuses specifically on the most abundant trait. More generally, the concept of dysbiosis is vast and cannot be reduced to a single quantitative measure valid for all chronic respiratory diseases and all populations [55, 56].

On the other hand, using random effects models, we highlighted a strong heterogeneity between the studies, which makes comparisons challenging and limits our conclusions with the available data (Fig. 2). Intra-individual variability [57], inter-individual variability, differences in study populations, samples, clustering approaches [58], microbiome sampling techniques and protocols, and other study characteristics are potential sources of heterogeneity. Using FAMD [18], we conducted an exploratory data analysis allowing us to analyze the similarity/dissimilarity of the studies according to certain factors at the origin of the heterogeneity reported in the papers (the sample size of cases and controls, the type of samples, the samples origin continent, the NGS sequencing method, the use of rarefaction analysis, the taxonomic level used). Our use of FAMD in exploring bias in meta-analyses is innovative and was completed with ANOVA tests. However, these analyses are limited by the small number of studies included (n=24, Fig. 1), which, in particular, did not allow us to group studies according to the type of samples.

We did not identify any evident links between alpha-diversity results and the studies’ characteristics or quality (Fig. 3), even if the sample size, the use of normalization method such as rarefaction, and the suitableness of collecting sputa as sampling method appeared to be notable study characteristics. As there was an important imbalance between studies in term of participants, studies with larger numbers of participants (and therefore smaller SD and wider $95\%$CI) have a greater weight in the meta-analysis results. This may have impacted the result in situations were few studies were available. These results reinforce the need to standardize the protocol to analyze the respiratory microbiota, additionally respiratory flora appears to be not limited to bacterial flora but also composed of viral and fungal floras [43].

In order to limit the great heterogeneity present in this field, we focused on the most widespread measures in the literature (the Shannon, Chao1, and Simpson alpha-diversity indices of the bacterial component); on studies presenting at least one case group and at least one control group (defined on the basis of the disease); on adults; and on studies using techniques that are not too far apart. As a consequence of this choice, some relatively large observational studies and trials providing interesting data and results were not included since they did not reach the study inclusion criteria. This was the case for the BLESS and CAMEB cohorts on non-cystic fibrosis bronchiectasis and the U-BIOPRED cohort on asthma [59–62]. Analyses of the CAMEB cohort focused either on analysis of the mycobiome exclusively [60] or on analysis of the entire microbiome (including bacteria, viruses and fungi) [61]. Shannon diversity was calculated but on the basis of renormalized and concatenated data, in agreement with the objective of the study (to assess the whole microbiome) but not with the objective of our meta-analysis (to assess the alpha-diversity of the bacterial microbiome). The study on BLESS [59] performed an analysis of the Bray Curtis index and relative abundances of specific species (Pseudomonas aeruginosa and Haemophilus influenza), but no analysis of alpha-diversity indices is provided. Finally, a recent publication on the U-BIOPRED cohort [62] measured alpha-diversity indices. However, the objective of this study was to identify phenotypes or clusters of severe asthma based on sputum microbiome profiles and to assess their stability after one year of follow-up. Given the longitudinal aspect and the absence of a control group in this study, it seemed inappropriate to include it in our meta-analysis. The lack of important studies (either by large sample size or by other quality criteria) is therefore a limitation of our study. Our study does not provide an overview of knowledge in the field, but rather a review of knowledge provided by studies based on the most commonly used criteria.

A second limitation of our meta-analysis is that it relies on the estimation of means and SD for studies reporting only quartiles. We used a method that does not rely on normality [13], but these estimates may be sensitive to small sample sizes, which are common in studies of respiratory microbiota. We argue for more data available in articles, additional files, or researchers’ web pages that would allow for better comparison of studies, with more reliable data.

## Conclusions

To conclude, while it is well admitted that high gut microbiota diversity is associated to health, the present meta-analysis showed that the current available knowledge and data do not allow us to extrapolate this result to the respiratory microbiota. Even though we noted some trends toward the same conclusion for some diseases (e.g. healthy vs CF), we also showed that it is not the case for all diseases (eg. healthy vs asthma or COPD). It is moreover difficult to perform comparisons across studies, because of the high heterogeneity detailed using random effects models. Knowledge on respiratory microbiota and health is under construction, and for the moment, it seems that the measurement of alpha-diversity isn’t enough to fully understand the link between microbiota and health, excepted in CF context which represents the most studied chronic respiratory disease with consistent data to link alpha-diversity and lung function [22] and ours. Whether differences in respiratory microbiota profiles have an impact on chronic respiratory disease symptoms and/or evolution deserves further exploration. Finally, as methods and practices tend to homogenize in gut microbiota analysis, we may expect the same evolution will happen soon to respiratory microbiota analysis and will help us to establish comparison between studies.

## Supplementary Information

**Additional file 1.** Additional file table and figures.

## Data Availability

All data collected during this study as well as the R codes developed to analyze and visualize the data are available on https://github.com/mavalosf.

## Abbreviations

NGS: Next generation sequencing
COPD: Chronic obstructive pulmonary disease
CF: Cystic fibrosis
NCFB: Non-Cystic fibrosis bronchiectasis
PRISMA: Preferred reporting Items for systematic reviews and meta-analyses
MOOSE: Meta-analysis of observational studies in epidemiology
PROSPERO: International Prospective Register of Systematic Reviews
DNA: DeoxyriboNucleic acid
SD: Standard deviation
ANOVA: Confidence Intervals at confidence level $95\%$ ($95\%$ IC) Analysis of variance
FAMD: Factor analysis of mixed data
OTU: Operational Taxonomic Unit
ASV: Amplicon sequence variant
BAL: Bronchoalveolar lavage
LA: Lower airways
UA: Upper airways
T-RFLP: Terminal Restriction Fragment Length Polymorphism
