# Supplementary material for: The respiratory microbiota alpha-diversity in chronic lung diseases: first systematic review and meta-analysis
Source: Respir Res. 2022 Aug 23;23:214. doi: 10.1186/s12931-022-02132-4 (PMC9396807; doi:10.1186/s12931-022-02132-4)
Supplement: Supplementary file 1 — Additional file 1. Additional file table and figures. [file 12931_2022_2132_MOESM1_ESM.pdf]

Additional Files

Disease	Variable	ANOVA p-value
Asthma	Sample	0.81
	Continent	0.01
	Sequencing	0.95
	Rarefaction	0.82
	Taxonomic level	0.06
	ASV method	0.48
	Score	0.55
COPD	Sample	0.75
	Continent	0.10
	Sequencing	0.13
	Rarefaction	0.29
	Taxonomic level	0.20
	ASV method	0.36
	Score	0.70
CF	Sample	0.09
	Continent	0.30
	Sequencing	0.02
	Rarefaction	0.28
	Taxonomic level	0.28
	ASV	0.13
	Score	0.31
All diseases	Sample	0.12
	Continent	0.59
	Sequencing	0.12
	Rarefaction	0.53
	Taxonomic level	0.02
	ASV	0.29
	Score	0.44

Table 5 ANOVA p-values to assess the effect of characteristics on the difference in Shannon values between cases and controls. A random-effects model is applied per characteristic (no adjustment for other characteristics or for the comparison group is performed because of the small number of studies).

Figure 4 FAMD biplot. Studies are represented by blue points (Byu17 [46] Car13 [27], Cob15 [45], Den16 [29], Ein16 [39], Erb11 [37], Fei17 [25], Gol13 [28], Hua20 [34], Kim17 [4], Lee18 [20], Li17 [32], Liu20 [31], Mar13 [33], Mil15 [41], Mun16 [35], Nar17 [44], Par14 [36], Ple19 [42], Pra12 [38], Sor20 [43], Sve17 [30], Wan20 [21], Wil09 [23]), continuous variables (cases and controls group sizes) are represented by red vectors and the modalities of the categorical variables (type of samples: bronchoalveolar lavage (BAL), sputum, sputum (induced), lower airways (LA), upper airways (UA); the samples origin continent: Asia, America, or Europe; the NGS sequencing method: 454 pyrosequencing for Pyrosequencing such as 454 FLX (Roche©), Illumina for bridge amplification such as MiSeq or HiSeq (Illumina©), 454 pyrosequencing/Illumina for combined methods, PacBio/Illumina for long read sequencing such as PacBio (Pacific Biosciences©) combined with Illumina, Other for the other combinations; the use of rarefaction analysis: YES or NO/NC (no or not stated clearly); the taxonomic level used: OTU vs. Genus); the clustering method: OTU vs. ASV) are represented by black triangles. As AFDM algorithm works like principal component analysis (PCA) for quantitative variables and like multiple correspondence analysis (MCA) for qualitative variables, the contributing quantitative variables are highlighted using vectors (referring to the circle of correlations as in PCA) while the qualitative variables are represented according to their exact barycentre (referring to their contribution values in MCA); all the variables being normalized during the analysis to balance the influence of each set of variables.

Figure 5 FAMD biplot. Studies (Byu17 [46] Car13 [27], Cob15 [45], Den16 [29], Ein16 [39], Erb11 [37], Fei17 [25], Gol13 [28], Hua20 [34], Kim17 [4], Lee18 [20], Li17 [32], Liu20 [31], Mar13 [33], Mil15 [41], Mun16 [35], Nar17 [44], Par14 [36], Ple19 [42], Pra12 [38], Sor20 [43], Sve17 [30], Wan20 [21]) are colored by the size of the case group. The farther to the right, the larger the sample size.

Figure 6 FAMD biplot. Studies (Byu17 [46] Car13 [27], Cob15 [45], Den16 [29], Ein16 [39], Erb11 [37], Fei17 [25], Gol13 [28], Hua20 [34], Kim17 [4], Lee18 [20], Li17 [32], Liu20 [31], Mar13 [33], Mil15 [41], Mun16 [35], Nar17 [44], Par14 [36], Ple19 [42], Pra12 [38], Sor20 [43], Sve17 [30], Wan20 [21]) are colored by the size of the control group. The studies with the largest control sample size are located at the lower right.

Figure 7 FAMD biplot. Studies (Byu17 [46] Car13 [27], Cob15 [45], Den16 [29], Ein16 [39], Erb11 [37], Fei17 [25], Gol13 [28], Hua20 [34], Kim17 [4], Lee18 [20], Li17 [32], Liu20 [31], Mar13 [33], Mil15 [41], Mun16 [35], Nar17 [44], Par14 [36], Ple19 [42], Pra12 [38], Sor20 [43], Sve17 [30], Wan20 [21]) are colored by the type of sample. Studies based on BAL samples are mainly located at the top left of the graph, studies based on sputum (induced) samples are mainly located at the right, studies based on UA samples are mainly located at the top, studies based on LA samples are mainly located at the bottom left of the graph. Finally, studies based on sputum samples are mainly scattered in the center of the graph.

Figure 8 FAMD biplot. Studies (Byu17 [46] Car13 [27], Cob15 [45], Den16 [29], Ein16 [39], Erb11 [37], Fei17 [25], Gol13 [28], Hua20 [34], Kim17 [4], Lee18 [20], Li17 [32], Liu20 [31], Mar13 [33], Mil15 [41], Mun16 [35], Nar17 [44], Par14 [36], Ple19 [42], Pra12 [38], Sor20 [43], Sve17 [30], Wan20 [21]) are colored by the samples origin continent. Asian studies are clearly clustered in the upper right diagonal. American and European studies are mixed in the opposite direction.

Figure 9 FAMD biplot. Studies (Byu17 [46] Car13 [27], Cob15 [45], Den16 [29], Ein16 [39], Erb11 [37], Fei17 [25], Gol13 [28], Hua20 [34], Kim17 [4], Lee18 [20], Li17 [32], Liu20 [31], Mar13 [33], Mil15 [41], Mun16 [35], Nar17 [44], Par14 [36], Ple19 [42], Pra12 [38], Sor20 [43], Sve17 [30], Wan20 [21]) are colored by the NGS sequencing method. 454 pyrosequencing, for Pyrosequencing such as 454 FLX (Roche©), are clustered in the center; Illumina, for bridge amplification such as MiSeq or HiSeq (Illumina©), are mostly at the bottom left; 454 pyrosequencing/Illumina, for combined methods, is isolated at the top right; PacBio/Illumina, for long read sequencing such as PacBio (Pacific Biosciences©) combined with Illumina, is isolated at the bottom right; Other, for the other combinations, are clustered in the top center.

Figure 10 FAMD biplot. Studies (Byu17 [46] Car13 [27], Cob15 [45], Den16 [29], Ein16 [39], Erb11 [37], Fei17 [25], Gol13 [28], Hua20 [34], Kim17 [4], Lee18 [20], Li17 [32], Liu20 [31], Mar13 [33], Mil15 [41], Mun16 [35], Nar17 [44], Par14 [36], Ple19 [42], Pra12 [38], Sor20 [43], Sve17 [30], Wan20 [21]) are colored by the use of rarefaction analysis: YES or NO/NC (no or not stated clearly). Studies with rarefaction analysis are mainly grouped on the bottom right, the others are mainly grouped on the top left.

Figure 11 FAMD biplot. Studies (Byu17 [46] Car13 [27], Cob15 [45], Den16 [29], Ein16 [39], Erb11 [37], Fei17 [25], Gol13 [28], Hua20 [34], Kim17 [4], Lee18 [20], Li17 [32], Liu20 [31], Mar13 [33], Mil15 [41], Mun16 [35], Nar17 [44], Par14 [36], Ple19 [42], Pra12 [38], Sor20 [43], Sve17 [30], Wan20 [21]) are colored by the taxonomic level used (OTU vs. Genus). Studies at OTU level are mainly grouped on the bottom right, studies at genus level are mainly grouped on the top left.

Figure 12 FAMD biplot. Studies (Byu17 [46] Car13 [27], Cob15 [45], Den16 [29], Ein16 [39], Erb11 [37], Fei17 [25], Gol13 [28], Hua20 [34], Kim17 [4], Lee18 [20], Li17 [32], Liu20 [31], Mar13 [33], Mil15 [41], Mun16 [35], Nar17 [44], Par14 [36], Ple19 [42], Pra12 [38], Sor20 [43], Sve17 [30], Wan20 [21]) are colored by the clustering method: OTU (No ASV) vs. ASV. Only two studies (located at the bottom right of the graph, not isolated) used a ASV-based approach.

Figure 13 FAMD biplot vs. quality score. Studies (Byu17 [46] Car13 [27], Cob15 [45], Den16 [29], Ein16 [39], Erb11 [37], Fei17 [25], Gol13 [28], Hua20 [34], Kim17 [4], Lee18 [20], Li17 [32], Liu20 [31], Mar13 [33], Mil15 [41], Mun16 [35], Nar17 [44], Par14 [36], Ple19 [42], Pra12 [38], Sor20 [43], Sve17 [30], Wan20 [21]) are colored by the sum of expert assessments. The study rated with a low quality score is fairly isolated in the upper left. However, the other studies are mixed together. Their arrangement in the graph cannot be interpreted in terms of quality score.

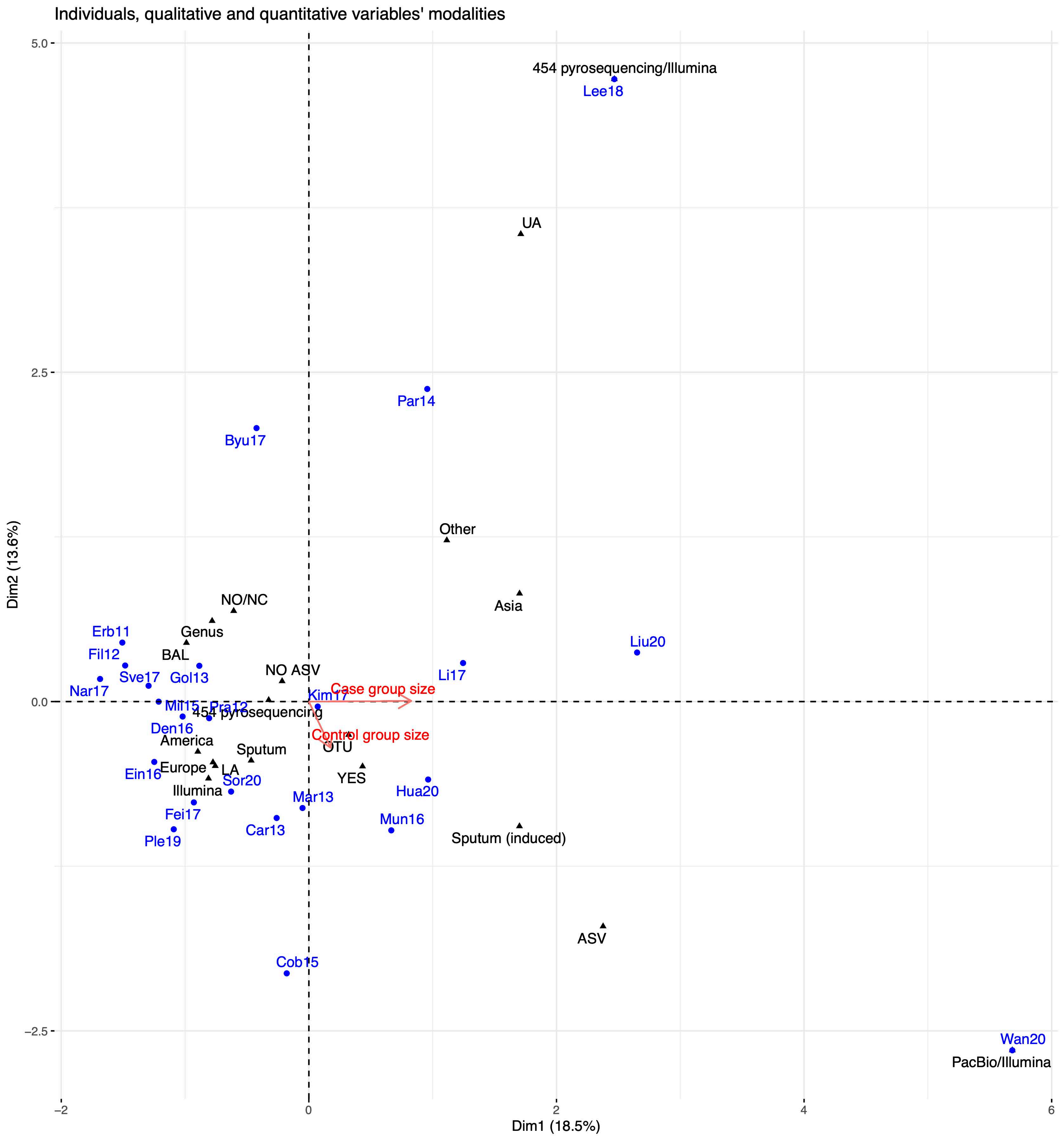

Individuals, qualitative and quantitative variables' modalities
Dim1 (18.5%)
Dim2 (13.6%)
454 pyrosequencing/Illumina
Lee18
UA
Par14
Byu17
Other
Asia
NO/NC
Genus
Erb11
Fil12
BAL
Liu20
Li17
Sve17
Gol13
NO ASV
Nar17
Case group size
Kim17
Mil15
Pra12
454 pyrosequencing
Den16
America
Control group size
Sputum
OTU
Europe
LA
Ein16
Sor20
YES
Illumina
Hua20
Mar13
Fei17
Mun16
Car13
Ple19
Sputum (induced)
ASV
Cob15
Wan20
PacBio/Illumina

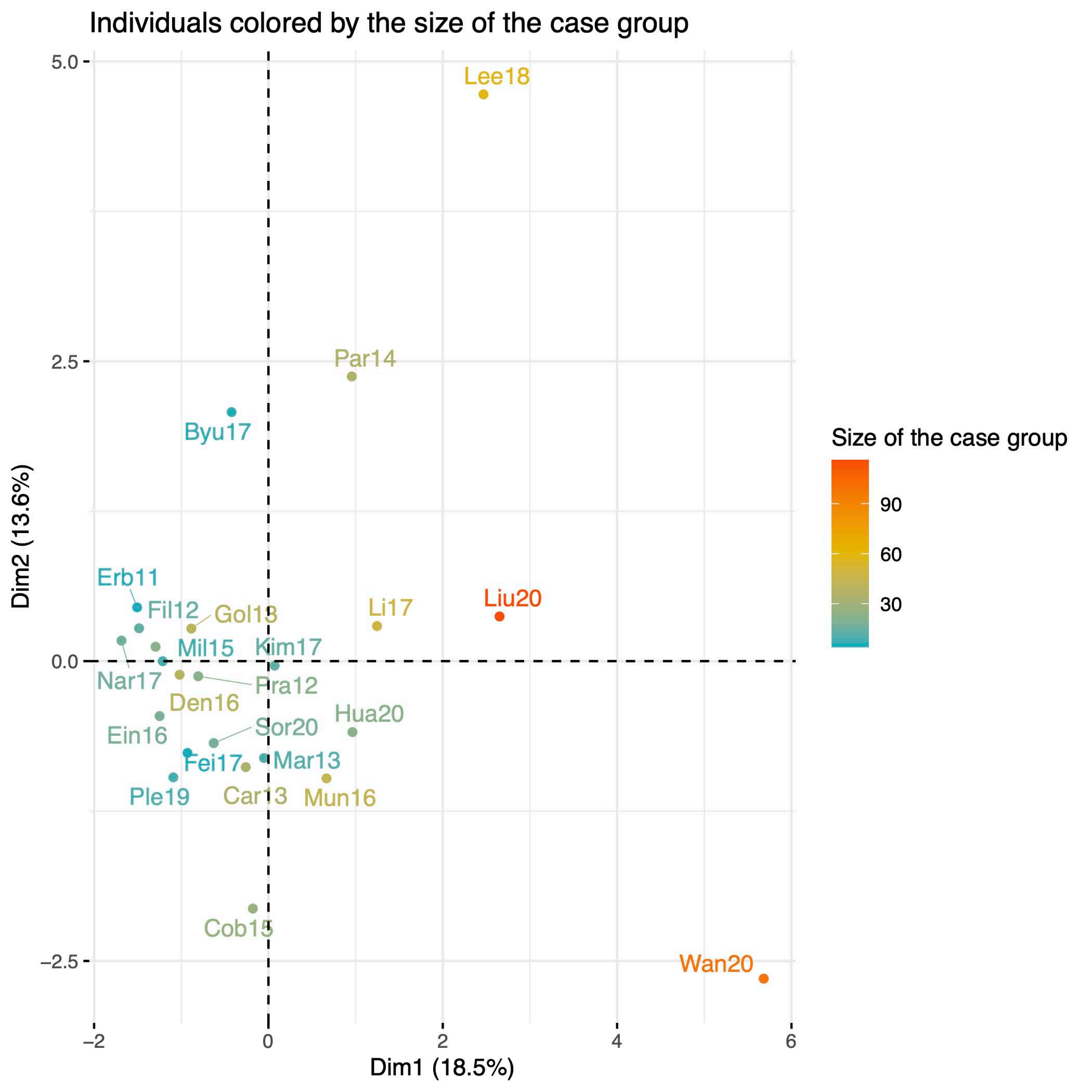
Individuals colored by the size of the case group
Size of the case group
90
60
30
Dim1 (18.5%)
Dim2 (13.6%)
5.0
2.5
0.0
−2.5
−2
0
2
4
6
Lee18
Par14
Byu17
Erb11
Fil12
Gol13
Li17
Liu20
Mil15
Kim17
Nar17
Pra12
Den16
Hua20
Ein16
Sor20
Fei17
Mar13
Ple19
Car13
Mun16
Cob15
Wan20

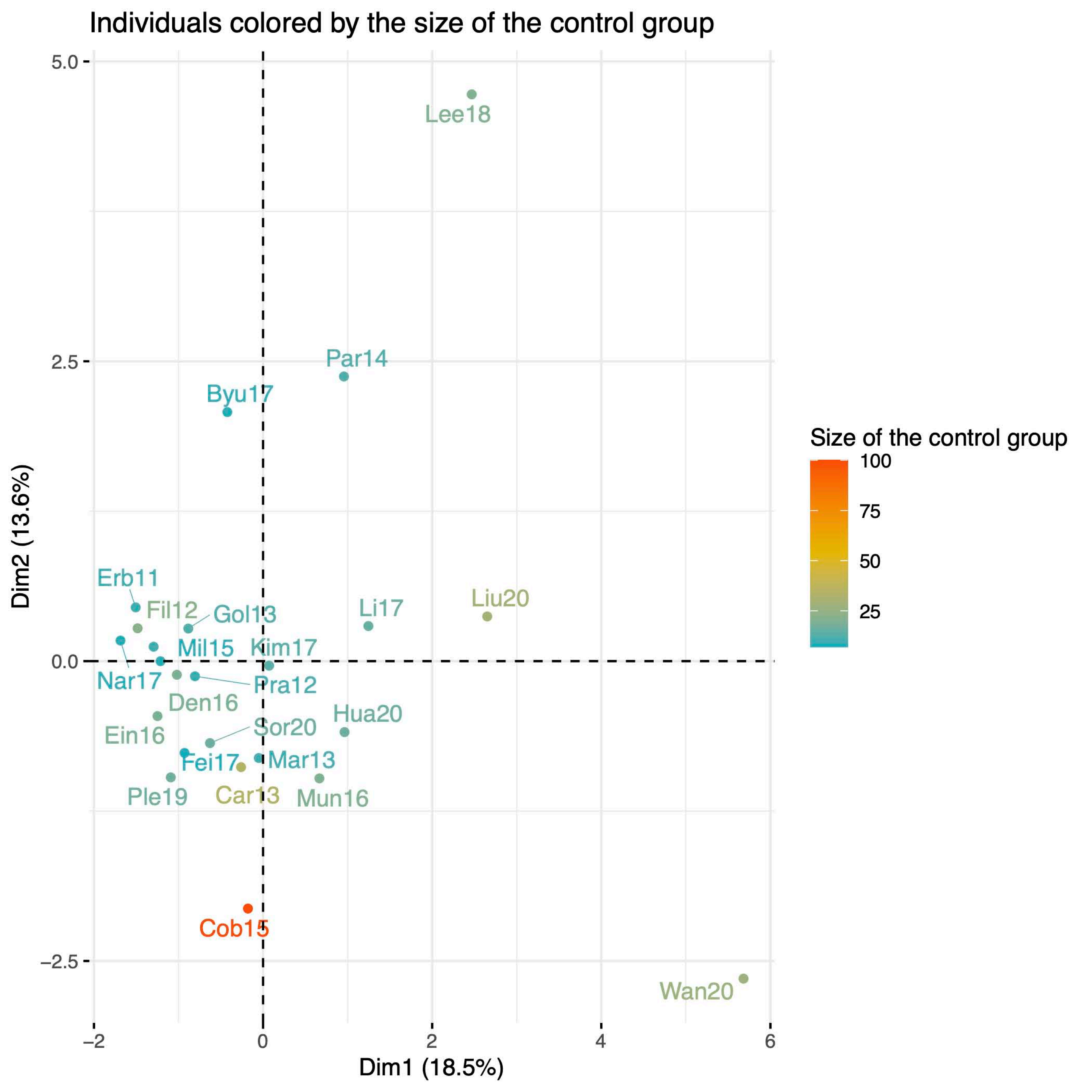
Individuals colored by the size of the control group
Lee18
Par14
Byu17
Erb11
Fil12
Gol13
Li17
Liu20
Mil15
Kim17
Nar17
Pra12
Den16
Hua20
Sor20
Ein16
Fei17
Mar13
Ple19
Car13
Mun16
Cob15
Wan20
Size of the control group
100
75
50
25
Dim2 (13.6%)
Dim1 (18.5%)
5.0
2.5
0.0
−2.5
−2
0
2
4
6

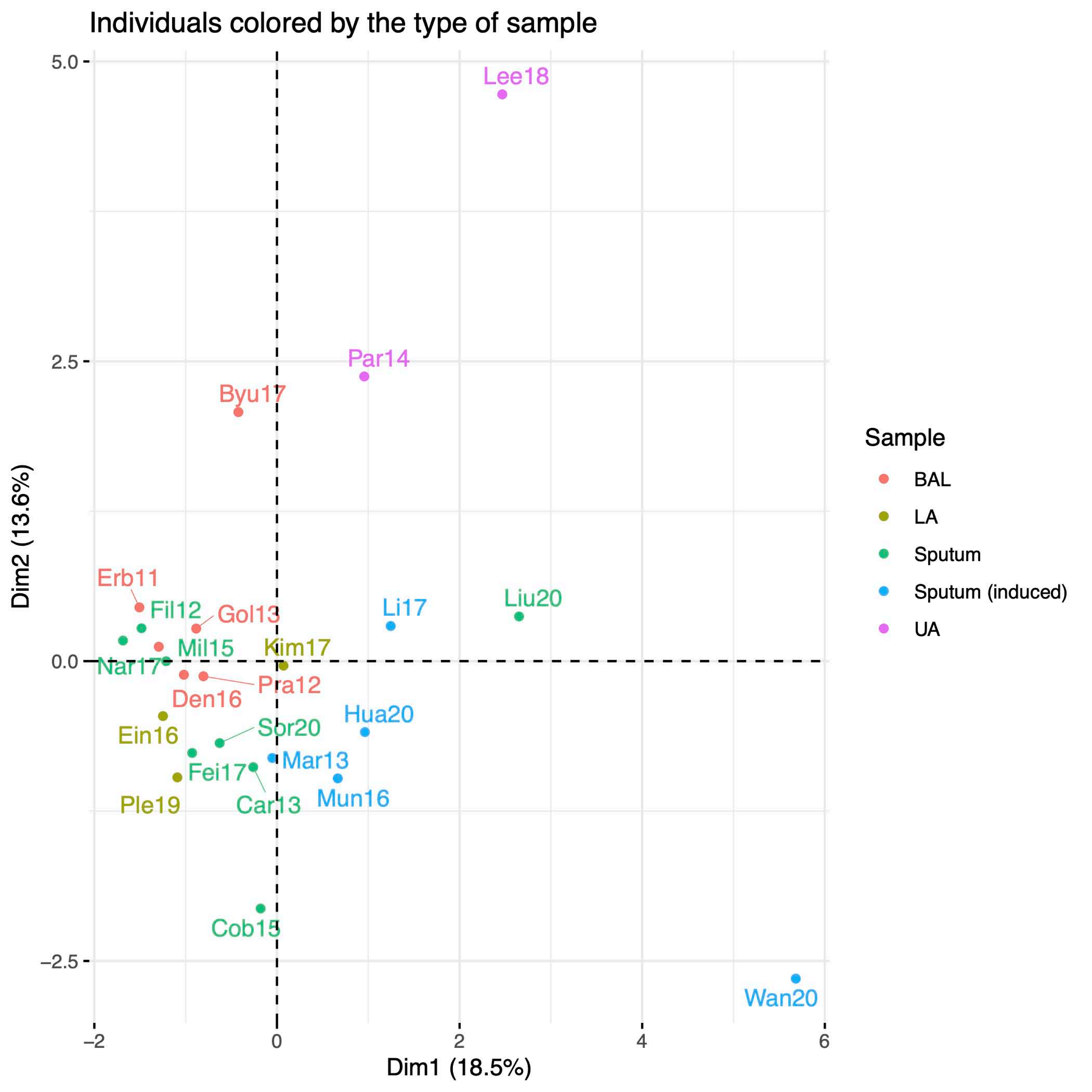
Individuals colored by the type of sample
Dim1 (18.5%)
Dim2 (13.6%)
5.0
2.5
0.0
−2.5
−2
0
2
4
6
Lee18
Par14
Byu17
Erb11
Fil12
Gol13
Mil15
Kim17
Nar17
Pra12
Den16
Li17
Liu20
Hua20
Ein16
Sor20
Mar13
Fei17
Mun16
Ple19
Car13
Cob15
Wan20
Sample
BAL
LA
Sputum
Sputum (induced)
UA

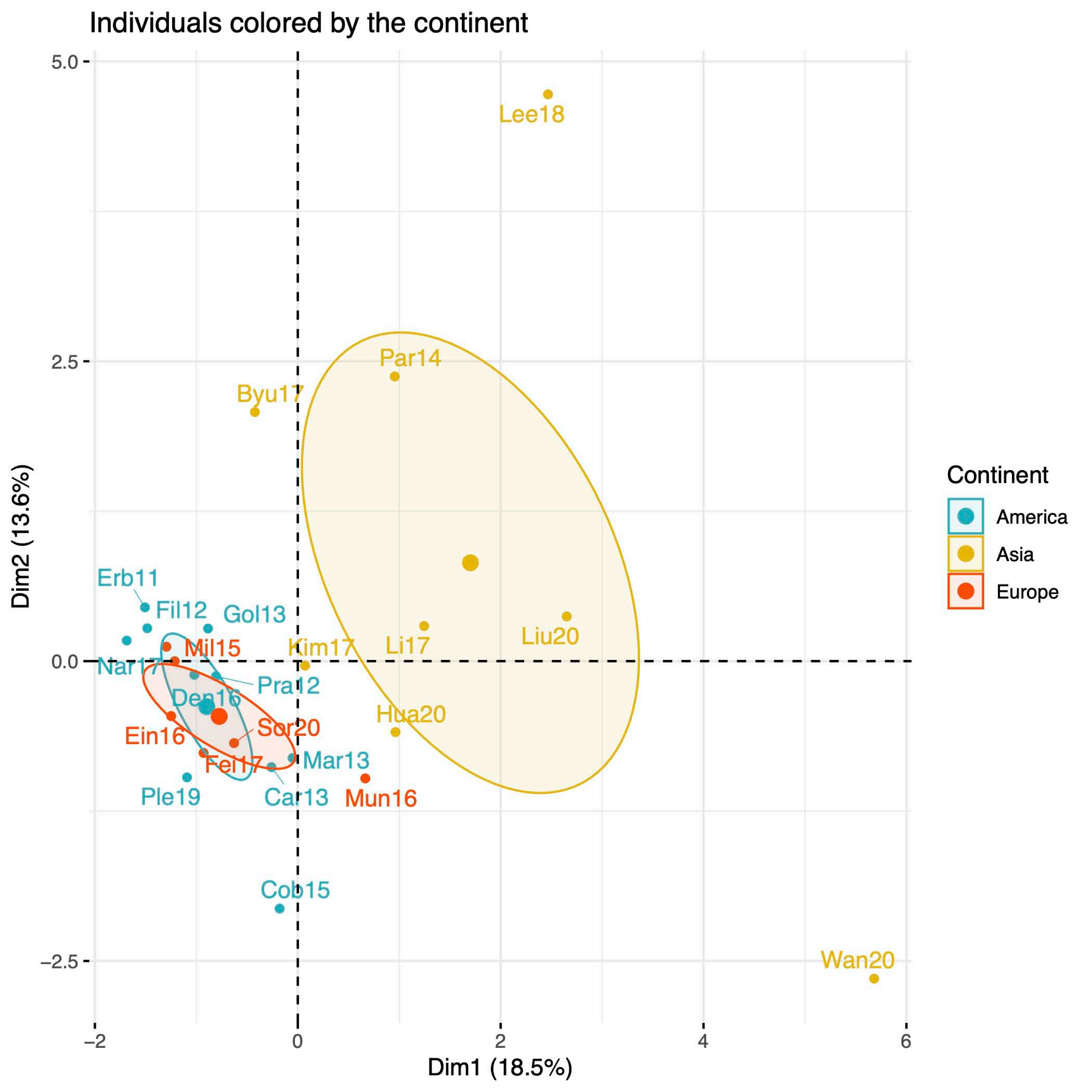

Individuals colored by the continent
Dim1 (18.5%)
Dim2 (13.6%)
5.0
2.5
0.0
−2.5
−2
0
2
4
6
Lee18
Par14
Byu17
Erb11
Fil12
Gol13
Mil15
Kim17
Li17
Liu20
Nar17
Pra12
Den16
Sor20
Ein16
Hua20
Fei17
Mar13
Ple19
Car13
Mun16
Cob15
Wan20
Continent
America
Asia
Europe

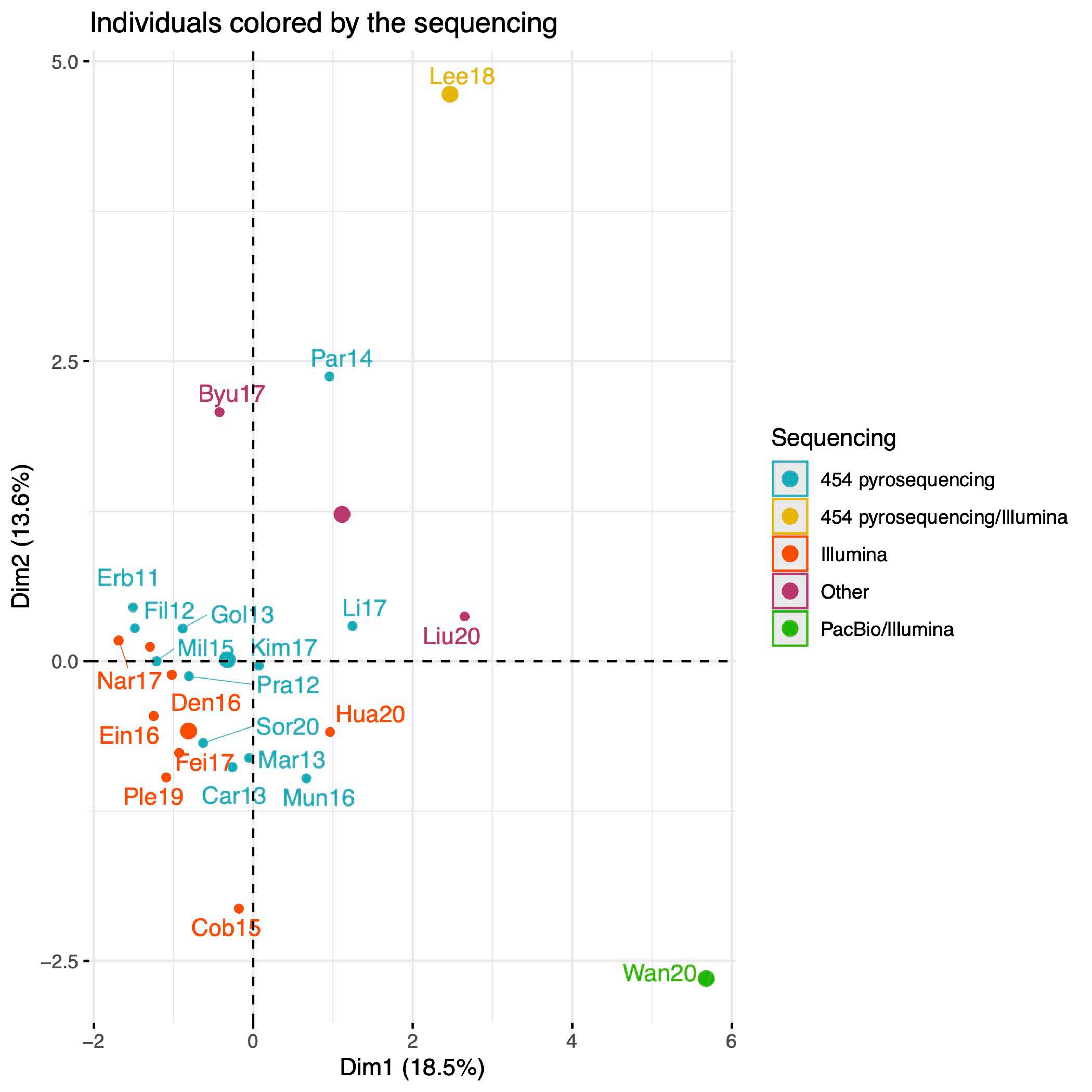

Individuals colored by the sequencing
Dim1 (18.5%)
Dim2 (13.6%)
5.0
2.5
0.0
−2.5
−2
0
2
4
6
Lee18
Par14
Byu17
Erb11
Fil12
Gol13
Li17
Liu20
Mil15
Kim17
Nar17
Pra12
Den16
Hua20
Sor20
Ein16
Fei17
Mar13
Ple19
Car13
Mun16
Cob15
Wan20
Sequencing
454 pyrosequencing
454 pyrosequencing/Illumina
Illumina
Other
PacBio/Illumina

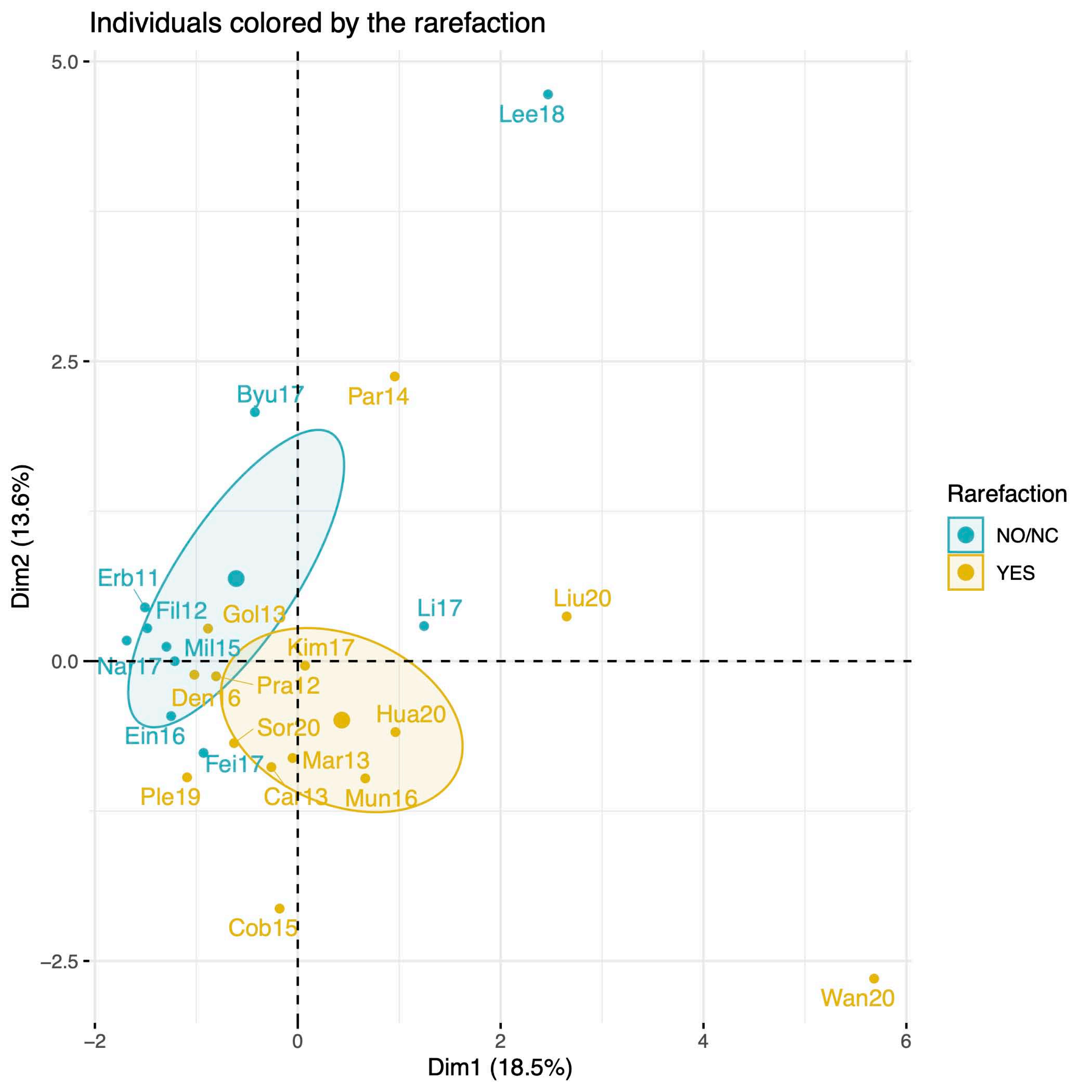
Individuals colored by the rarefaction
Dim2 (13.6%)
Dim1 (18.5%)
5.0
2.5
0.0
−2.5
−2
0
2
4
6
Lee18
Byu17
Par14
Erb11
Fil12
Gol13
Li17
Liu20
Mil15
Kim17
Nar17
Pra12
Den16
Hua20
Sor20
Ein16
Mar13
Fei17
Ple19
Cal13
Mun16
Cob15
Wan20
Rarefaction
NO/NC
YES

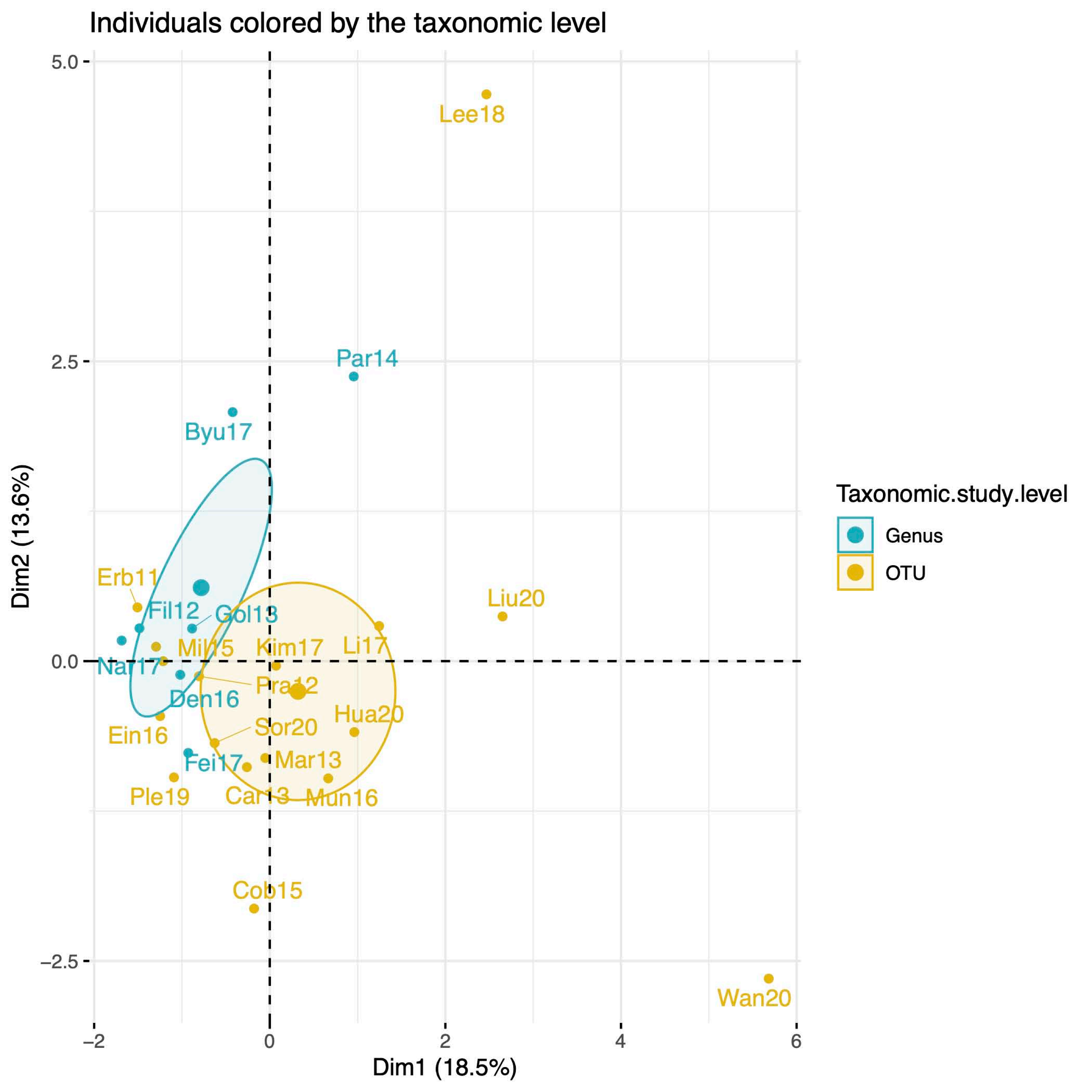

Individuals colored by the taxonomic level
Dim2 (13.6%)
Dim1 (18.5%)
5.0
2.5
0.0
−2.5
−2
0
2
4
6
Lee18
Par14
Byu17
Erb11
Fil12
Gol13
Mil15
Kim17
Li17
Liu20
Nar17
Pra12
Den16
Hua20
Ein16
Sor20
Mar13
Fei17
Ple19
Car13
Mun16
Cob15
Wan20
Taxonomic.study.level
Genus
OTU

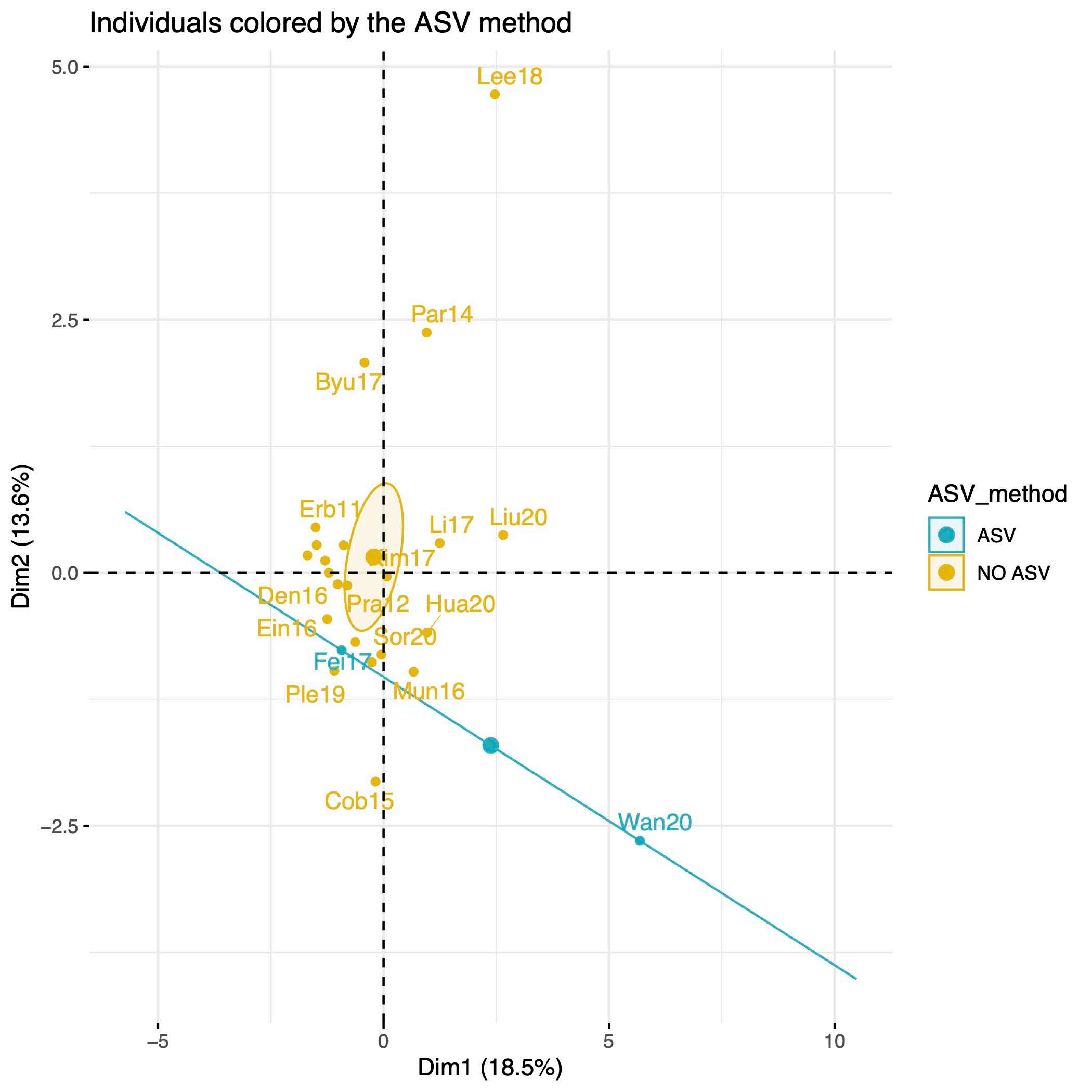
Individuals colored by the ASV method
5.0
2.5
0.0
−2.5
−5
0
5
10
Dim1 (18.5%)
Dim2 (13.6%)
Lee18
Par14
Byu17
Erb11
Li17
Liu20
Kim17
Den16
Pra12
Hua20
Ein16
Sor20
Fei17
Ple19
Mun16
Cob15
Wan20
ASV_method
ASV
NO ASV

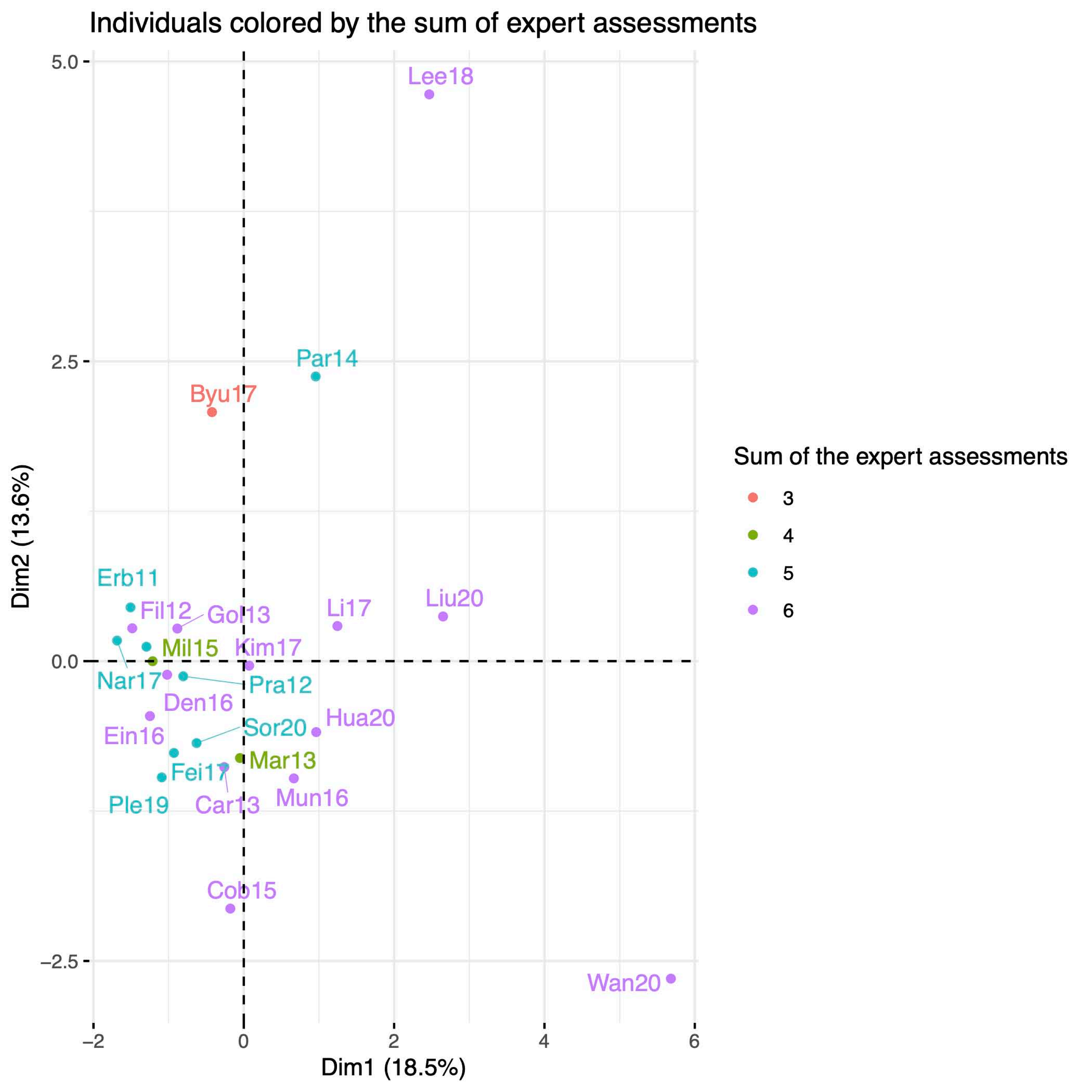
Individuals colored by the sum of expert assessments
Dim2 (13.6%)
Dim1 (18.5%)
5.0
2.5
0.0
−2.5
−2
0
2
4
6
Lee18
Par14
Byu17
Erb11
Fil12
Gol13
Li17
Liu20
Mil15
Kim17
Nar17
Pra12
Den16
Hua20
Sor20
Ein16
Mar13
Fei17
Mun16
Ple19
Car13
Cob15
Wan20
Sum of the expert assessments
3
4
5
6
